# Quality of Web-based Information for the 10 Most Common Fractures

**DOI:** 10.2196/ijmr.5767

**Published:** 2016-06-17

**Authors:** Muzammil Memon, Lydia Ginsberg, Nicole Simunovic, Bill Ristevski, Mohit Bhandari, Ydo Vincent Kleinlugtenbelt

**Affiliations:** ^1^ McMaster University Medical Student Hamilton, ON Canada; ^2^ McMaster University Undergraduate Life Science Student Hamilton, ON Canada; ^3^ McMaster University Department of Clinical Epidemiology and Biostatistics Hamilton, ON Canada; ^4^ McMaster University Division of Orthopaedic Surgery Hamilton, ON Canada; ^5^ Deventer Ziekenhuis Department of Orthopaedic and Trauma Surgery Deventer Netherlands

**Keywords:** fractures, quality, readability, patient, online, information, HONcode, DISCERN, Google

## Abstract

**Background:**

In today's technologically advanced world, 75% of patients have used Google to search for health information. As a result, health care professionals fear that patients may be misinformed. Currently, there is a paucity of data on the quality and readability of Web-based health information on fractures.

**Objectives:**

In this study, we assessed the quality and readability of Web-based health information related to the 10 most common fractures.

**Methods:**

Using the Google search engine, we assessed websites from the first results page for the 10 most common fractures using lay search terms. Website quality was measured using the DISCERN instrument, which scores websites as very poor (15-22.5), poor (22.5-37.5), fair (37.5-52.5), good (52.5-67.5), or excellent (67.5-75). The presence of Health on the Net code (HONcode) certification was assessed for all websites. Website readability was measured using the Flesch Reading Ease Score (0-100), where 60-69 is ideal for the general public, and the Flesch-Kincaid Grade Level (FKGL; −3.4 to ∞), where the mean FKGL of the US adult population is 8.

**Results:**

Overall, website quality was “fair” for all fractures, with a mean (standard deviation) DISCERN score of 50.3 (5.8). The DISCERN score correlated positively with a higher website position on the search results page (*r*^2^=0.1, *P*=.002) and with HONcode certification (*P*=.007). The mean (standard deviation) Flesch Reading Ease Score and FKGL for all fractures were 62.2 (9.1) and 6.7 (1.6), respectively.

**Conclusion:**

The quality of Web-based health information on fracture care is fair, and its readability is appropriate for the general public. To obtain higher quality information, patients should select HONcode-certified websites. Furthermore, patients should select websites that are positioned higher on the results page because the Google ranking algorithms appear to rank the websites by quality.

## Introduction

In today's technologically advanced world, patients are increasingly using the Internet as their first source of health information [1,2]. Given the increased accessibility of the Internet, 75% of patients have used Google in the past to search for health information [3-6]. Although this is a large step toward shared decision making, health care professionals have expressed fear of patients becoming misinformed, potentially leading to adverse health outcomes. Furthermore, physicians are burdened, as they must clarify inaccuracies in the patients’ understanding of their illness or details surrounding treatment options [7]. Their concern is supported by several individual studies that have demonstrated that the quality and readability of health information websites is low and variable, as well as a systematic review by Eysenbach et al, evaluating studies assessing the quality of Web-based health information, which showed that quality was a problem [8-10]. Furthermore, these studies discovered that websites that are granted Health on the Net code (HONcode) certification for being high quality are just as variable as noncertified websites [11]. However, little is known about the usefulness of the information on the Internet for patients concerning fracture care. To address the usefulness of the information on the Internet for patients regarding fracture care, we determined the quality and readability of Web-based health information on the 10 most common fractures [12]. Therefore, the aims of this study are 2-fold: (1) to evaluate the quality of Web-based health information on fracture care and (2) to investigate the readability of this information.

## Methods

### Search Engine

The Google search engine was chosen for this study because 92% of patients who use the Internet as a resource for health information reported using Google [5]. The Google Chrome web browser was used. Web browsing data were deleted before each search was performed. Default search engine settings were used, producing 10 website results per search. The location settings on the search engine were set to Hamilton, Ontario, and the search was performed on March 23, 2015.

### Search Terms

We investigated the 10 most common fractures as they make up 83.8% (4990/5953) of all fractures [12]. We used lay search terms because it is known that patients are unsure of the meaning of orthopedic terms [13]. The search terms corresponding to each fracture type were: ‘‘broken wrist” (ie, distal radius fracture), “broken hand” (ie, metacarpal fracture), “broken hip” (ie, proximal femur fracture), “broken finger” (ie, finger phalanx fracture), “broken ankle” (ie, ankle fracture), “broken foot” (ie, metatarsal fracture), “broken shoulder” (ie, proximal humerus fracture), “broken elbow” (ie, proximal forearm fracture), “broken toe” (ie, toe phalanx fracture), and “broken collarbone” (ie, clavicle fracture).

### Inclusion and Exclusion Criteria

We used the first page of results for each search term because 92% of Google traffic is limited to the first page [14]. Websites were eligible for inclusion if they were (1) in English, (2) free to access, and (3) provided information on the fracture associated with the search term. Websites were excluded if they were primarily non–text-based (eg, YouTube), Web-based shopping sites, news articles, password protected, Google AdWords sponsored links, and forums.

### Quality Assessment

The quality of each website was scored using the DISCERN instrument (Multimedia Appendix 1). The DISCERN instrument is a validated questionnaire that assesses the reliability of websites and the quality of information on treatment choices [15]. This 16-question instrument is easy to use and can even be used by patients [15]. Each of the questions can receive a score from 1 to 5, corresponding to low and high quality, respectively. Questions 1-8 evaluate the reliability of the publication, questions 9-15 address the quality of information on treatment choices, and question 16 is an overall quality rating. Two independent raters who were medical and premedical students reached consensus on their DISCERN scores for each website (M.M. and L.G.). These 2 raters were supervised by an MD (Y.K.).

Different categorization ranges have arbitrarily been used in previous literature to interpret the total DISCERN score. We used categorical ranges, which have cut-off points set to the midpoint between each possible total DISCERN score to yield a more accurate interpretation of numeric total DISCERN scores. For example, if each question for one website scored a 1, the total DISCERN score would be 15, and if each question for a second website scored a 2, the total DISCERN score would be 30. The mean of the total DISCERN score of these 2 websites would be 22.5, which is what we set as the transition point between “very poor” and “poor.” Therefore, websites can score a total DISCERN score that is very poor (15-22.5), poor (22.5-37.5), fair (37.5-52.5), good (52.5-67.5), or excellent (67.5–75).

The presence of a HONcode certification seal was also assessed independently by the same 2 reviewers as the DISCERN rating (M.M. and L.G.). The Health on the Net Foundation provides HONcode certification to websites that demonstrate the intent to publish high-quality Web-based health information.

### Readability Assessment

**Figure 1:**
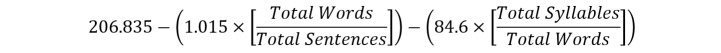
Fres formula.

**Figure 2:**
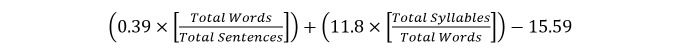
Fkgl formula.

The readability of each website was assessed using the Flesch Reading Ease Score (FRES) and the Flesch-Kincaid Grade Level (FKGL) [16]. See FRES formula in Figure 1. The FRES holds a value between 0-100 where passages scoring between 90 and 100 are easy to understand, passages scoring between 60 and 69 are ideal for the general public, and passages scoring under 30 are difficult to comprehend. See FKGL formula in Figure 2. The FKGL indicates the minimum US grade level required for a reader to comprehend a passage. The recommended FKGL for an adult patient in the United States is 6, whereas the mean FKGL of the US adult population is 8 [17,18]. To generate these scores, the website URLs were input into www.read-able.com, which automatically calculated these scores.

### Website Frequency and Affiliation

The frequency of websites among the top 3 search results for each search term was tabulated. Website affiliation was also tabulated into 5 categories including, Private Medical Company, Hospital or Clinic Network, Professional Medical Society, Governmental Organization, and Open Source Websites. Private Medical Companies included websites such as WebMD, which had no primary association with governmental or medical societies. Hospital or Clinic Networks included websites such as Mayo Clinic, which are run by large hospital networks and also smaller private clinics. Professional Medical Societies included the American Association of Orthopaedic Surgeons’ website, which were run by their respective societies. Governmental Organizations included websites such as Medline Plus, which are run by government organizations such as the US National Library of Medicine. Finally, open source websites included sites such as Wikipedia, which are freely editable by its users.

### Statistics

SPSS 20.0 statistics software (SPSS Inc, Chicago, IL, USA) was used to conduct all statistical analyses. Inter-rater agreement was assessed using weighted Kappa for ordinal data. Agreement was categorized a priori as follows: κ of .61 or greater was considered substantial agreement; κ of .21-.60, moderate agreement; and κ of .20 or less, slight agreement. Linear regressions were conducted to determine the association between DISCERN score and website position on the search results page, to determine an association between readability scores (FRES and FKGL) and website position on the search results page and to determine the association between readability scores and total DISCERN scores. These tests yielded *r*^2^and *P* values. A logistic regression was conducted to determine the association between website position on the search results page and HONcode presence, which yielded an odds ratio and *P* value. One-way analysis of variances were conducted to determine variance between the mean DISCERN score of websites produced for different search terms (ie, different fracture types), to assess variance between readability scores for websites that resulted for different search terms, and to assess variance between the DISCERN score, FRES, and FKGL for the different website affiliation categories. Independent *t* tests were conducted to determine whether a difference existed between the DISCERN scores of questions 1-8 and questions 9-15, to determine if there was a statistical difference between the DISCERN scores of websites with and without HONcode certification, and to determine if there was a statistical difference between the FRES and FKGL of websites with and without HONcode certification. The sensitivities and specificities of HONcode accreditation to predict poor, fair, and good quality websites, based on the DISCERN score, were calculated. A chi-square test was conducted to determine the correlation between HONcode presence and the fracture types associated with the search terms and the Fisher’s exact test *P* value was used, as the expected count was less than 5 in more than 1 cell. A *P* ≤.5 was considered to be significant.

## Results

### Website Search Results

Each of the search terms for the 10 fracture types returned 10 results on the first page, totaling 100.0 websites that were assessed. Thirteen websites were excluded because they did not include information on the fracture type associated with the search term (5), they were news articles (4), they were duplicates (3), and one website was a forum. The remaining 87 websites were included for quality assessment, readability calculation, and assessment of HONcode presence. The Kappa among reviewers for website inclusion was 1.00 and the Kappa for DISCERN ratings was .94.

### DISCERN Scores and HONcode Accreditation

**Figure 3:**
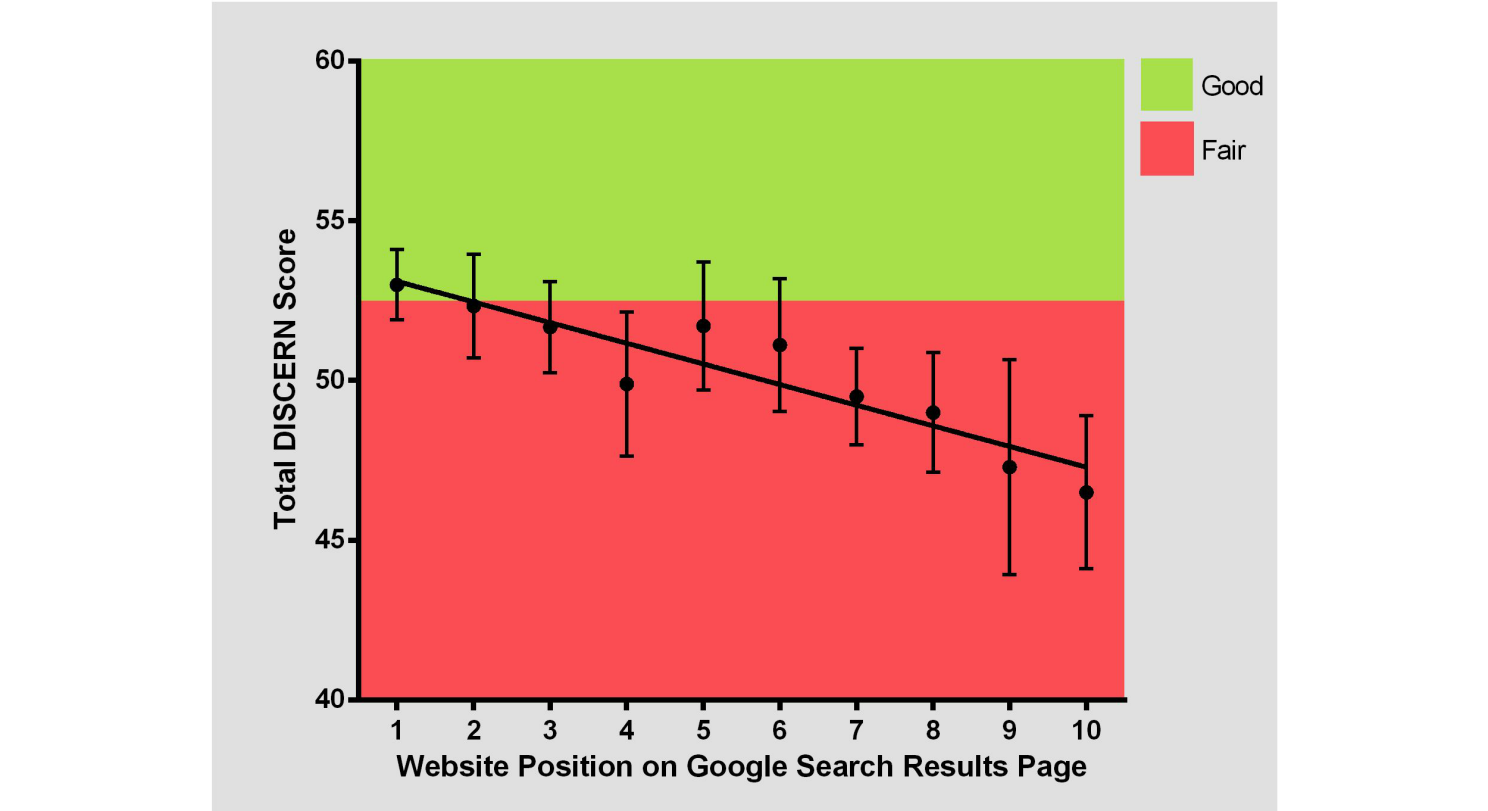
Correlation of total DISCERN score to website position on Google search results page (*r*^2^=0.104, *P*=.002).

Overall, the mean (standard deviation, SD) total DISCERN score for the 10 fractures was 50.3 (5.8), which is “fair” quality. “Broken hip” scored highest with a mean (SD) score of 55.1 (4.9), which is considered “good” quality. “Broken shoulder” and “broken finger” scored the lowest with mean (SD) scores of 46.5 (5.9) and 46.8 (4.9), respectively. However, the differences in the mean DISCERN scores of websites for each search term were statistically nonsignificant. The mean total DISCERN scores for each fracture type are shown in Figure 3. On average, DISCERN questions 4 and 12 received a mean score below 2, questions 2, 3, and 14 received a mean score above 4, and the remaining questions received a mean score between 2 and 4, inclusive. There was no statistical difference between questions 1-8 assessing website reliability, which had a mean (SD) DISCERN score of 3.6 (1.4) and questions 9-15 assessing quality of treatment information, which had a mean (SD) DISCERN score of 3.1 (1.5). The mean DISCERN scores for each question are shown in Multimedia Appendix. 1

**Figure 4:**
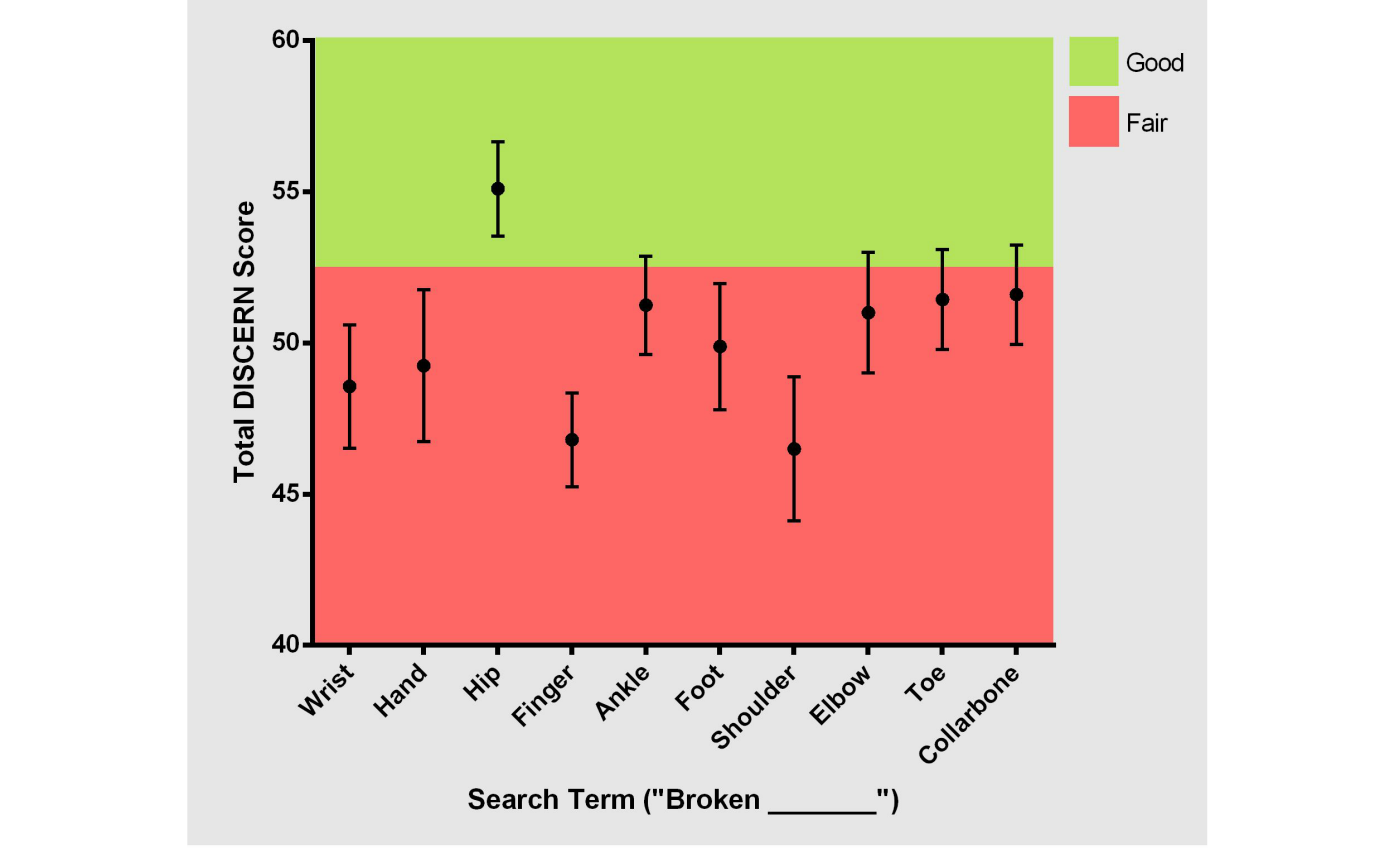
Distribution of total DISCERN score by search term.

There was a positive correlation between the DISCERN score and a higher website position on the search results page (*r*^2^=0.1, *P*=.002). As shown in Figure 4, websites positioned higher on the search results page had a higher total DISCERN score than websites positioned lower on the search results page.

With regard to HONcode accreditation, 49% (43/87; range 30%-75%) of websites displayed the HONcode certification seal. Furthermore, the DISCERN score demonstrated a positive correlation with HONcode accreditation, where the total DISCERN scores of HONcode accredited websites, which had a mean (SD) total DISCERN score of 52.0 (4.9), were statistically higher (*P*=.007) than the total DISCERN scores of websites that were not HONcode certified, which had a mean (SD) total DISCERN score of 48.8 (6.2) (Table 1). Specifically, the sensitivity and specificity (95% CI) of the HONcode accreditation was 0 (0-0.80) and 0.49 (0.38-0.60), respectively, for poor quality websites, 0.40 (0.27-0.56) and 0.40 (0.25-0.57), respectively, for fair quality websites, and 0.63 (0.46-0.78) and 0.61 (0.46-0.74), respectively, for good quality websites. The sensitivity and specificity of the HONcode accreditation for very poor and excellent quality websites was not calculated, as there were no websites within our data that ranked in those quality categories. With regard to website ranking, there was a positive correlation between a higher website position on the first Google results page and the presence of HONcode accreditation on the websites (odds ratio=0.723, *P<*.001) (Table 2). However, there was no significant correlation between the presence of HONcode accreditation on websites and the search term used (Tables 3 and 4).

**Table 1:** Statistical output of independent 2-sample *t*-test comparing HONcode accreditation and DISCERN score.

		Levene's test for equality of variances				*t*-test for equality of means				
		F	Sig.	*t*	df	Sig. (2 tailed)	Mean difference	Std. error difference	95% CI of the difference	
									Lower	Upper
DISCERN score	Equal variances assumed	2.714	.103	2.853	85	.005	3.40962	1.19493	1.03378	5.78546
	Equal variances not assumed			2.861	81.231	.005	3.40962	1.19163	1.03876	5.78048

**Table 2:** Statistical output of logistic regression comparing HONcode accreditation and website position on the search results page.

Source	DF	Adjusted deviation	Adjusted mean	Chi-square	*P* value
Regression	1	15.83	15.834	15.83	<.001
Website position	1	15.83	15.834	15.83	<.001
Error	85	104.76	1.232		
Total	86	120.60			

	Odds ratio	95% CI			
Website position	0.7231	0.6075-0.8606			

**Table 3:** Statistical output of R×C contingency table comparing HONcode accreditation and search term used for each fracture type.

		Search Term										Total
		Broken wrist	Broken hand	Broken hip	Broken finger	Broken ankle	Broken foot	Broken shoulder	Broken elbow	Broken toe	Broken collarbone	
HONcode accreditation	No	5	4	6	4	4	2	4	4	4	7	44
	Yes	4	4	4	6	4	6	2	5	5	3	43
Total		9	8	10	10	8	8	6	9	9	10	87

**Table 4:** Statistical output of chi-square test comparing HONcode accreditation and search term used for each fracture type.

	Value	df	Asymp. sig. (2 sided)	Exact sig. (2 sided)	Exact sig. (1 sided)	Point probability
Pearson chi-square	5.389^a^	9	.799	.819		
Likelihood ratio	5.546	9	.784	.817		
Fisher's exact test	5.491			.820		
Linear-by-linear association	.056^b^	1	.813	.829	.421	.028
N of valid cases	87					

^a^Seventeen cells (85.0%) have expected count less than 5. The minimum expected count is 2.97.

^b^The standardized statistic is −.236.

### Readability Scores

The mean (SD) FRES for all websites was 62.2 (9.1), which is ideal for the general public. The fracture types that had a mean (SD) below the range of scores ideal for the general public included “broken hip,” 59.3 (6.2); “broken shoulder,” 51.9 (8.6); “broken elbow,” 55.6 (13.8); and “broken collarbone,” 59.7 (11.5). The mean (SD) FKGL for all websites was 6.7 (1.6), which is greater than 6, the recommended reading level for the general public, but lower than 8, which is the mean FKGL of an average US adult. The only fracture type above the mean FKGL of a US adult was broken shoulder, with a mean (SD) of 8.1 (1.0). There was no statistically significant correlation between the FRES or FKGL and (1) the position of websites on the search results page, (2) the DISCERN score, and (3) the presence of HONcode certification (Tables 5 and 6). Furthermore, there was no statistically significant difference between the FRES or FKGL of the websites that resulted from the different search terms.

### Website Frequency and Affiliation

The frequency of the websites from the first 3 search results for all search terms in order of popularity was 6 of 30 (20%) for OrthoInfo, 6 of 30 (20%) for eMedicineHealth, 4 of 30 (13.3%) for WebMD, 3 of 30 (10%) for MedicineNet, 3 of 30 (10%) for Mayo Clinic, 1 of 30 (3.3%) for Drugs, 1 of 30 (3.3%) for Healthline, 1 of 30 (3.3%) for Wikipedia, 1 of 30 (3.3%) for Boots WebMD, 1 of 30 (3.3%) for Cleveland Clinic, 1 of 30 (3.3%) for Physio Works, 1 of 30 (3.3%) for Foot Health Facts, and 1 of 30 (3.3%) for National Health Service (Table 7) [19-30].

**Table 5:** Statistical output of independent 2-sample *t*-test comparing HONcode accreditation and Flesch Reading Ease Score (FRES)

		Levene's test for equality of variances				*t*-test for equality of means				
		F	Sig.	*t*	df	Sig. (2 tailed)	Mean difference	Std. error difference	95% CI of the difference	
									Lower	Upper
FRES	Equal variances assumed	.253	.617	−1.271	85	.207	−2.46131	1.93718	−6.31293	1.39031
	Equal variances not assumed			−1.271	84.999	.207	−2.46131	1.93656	−6.31171	1.38909

**Table 6:** Statistical output of independent 2-sample *t*-test comparing HONcode accreditation and Flesch-Kincaid Grade Level (FKGL)

		Levene's test for equality of variances				*t*-test for equality of means				
		F	Sig.	*t*	df	Sig. (2 tailed)	Mean difference	Std. error difference	95% CI of the difference	
									Lower	Upper
FKGL	Equal variances assumed	.589	.445	1.373	85	.173	.47156	.34339	−.21118	1.15431
	Equal variances not assumed			1.370	81.526	.174	.47156	.34412	−.21306	1.15619

**Table 7:** Frequency of websites from first 3 search results for all search terms.

Website title	Frequency (n=30)	Percentage	DISCERN, mean ± SD (range)	FRES, mean ± SD (range)	FKGL, mean ± SD (range)
OrthoInfo	6	20	52.2±5.0 (47.0-57.0)	57.9±7.0 (44.7-63.8)	7.2±0.8 (6.7-8.8)
eMedicineHealth	6	20	53.3±1.5 (52.0-55.0)	59.6±4.4 (55.8-64.5)	8.1±0.8 (7.2-8.6)
WebMD	4	13.3	50.3±2.5 (47-53)	65.9±2.5 (64.1-69.6)	5.7±0.5 (5-6.3)
MedicineNet	3	10	53.3±1.5 (52-55)	59.6±4.4 (55.8-64.5)	8.1±0.8 (7.2-8.6)
Mayo Clinic	3	10	56.0±1 (55.0-57.0)	56.9±6.3 (49.7-61.5)	6.9±0.4 (6.6-7.3)
Drugs	1	3.3	55.0	75.7	4.7
Healthline	1	3.3	56.0	65.9	5.7
Wikipedia	1	3.3	60.0	47.7	8.5
Boots WebMD	1	3.3	45.0	69.0	5.5
Cleveland Clinic	1	3.3	45.0	47.5	7.7
Physio Works	1	3.3	45.0	57.7	6.8
Foot Health Facts	1	3.3	45.0	58.1	9.1
National Health Service	1	3.3	52.0	75.6	4.5

**Table 8:** Distribution of website affiliation for all search results.

Website affiliation	Frequency (n=87)	Percentage	DISCERN, mean ± SD (range)	FRES, mean ± SD (range)	FKGL, mean ± SD (range)
Private Medical Company	39	44.8	50.0±4.9 (39.0-57.0)	60.1±13.1 (-2.7-78.4)	7.2±2.4 (4.5-17.0)
Hospital or Clinic Network	16	18.4	50.2±8.4 (37.0-60.0)	58.0±9.7 (31.4-65.4)	7.1±1.2 (5.5-9.9)
Professional Medical Society	16	18.4	49.6±5.0 (42.0-57.0)	60.6±6.3 (44.7-68.5)	6.4±1.0 (3.8-7.5)
Governmental Organization	9	10.3	52.6±4.6 (44.0-62.0)	66.8±7.7 (46.7-75.6)	5.8±1.1 (4.5-8.5)
Open Source Websites	7	8.0	48.0±9.1 (37.0-60.0)	66.5±13.3 (47.7-79.1)	5.7±1.9 (4.2-8.5)

With regard to website affiliation, 39 of 87 (44.8%) websites were from a Private Medical Company, 16 of 87 (18.4%) were from a Hospital or Clinic Network, 16 of 87 (18.4%) were from a Professional Medical Society, 9 of 87 (10.3%) were from a Governmental Organization, and 7 of 87 (8.0%) were from Open Source Websites (Table 8). Furthermore, there were no significant differences in the DISCERN scores, FRES, and FKGL values between the different website affiliation categories.

## Discussion

The aim of this study was to elucidate the quality of Web-based health information on the 10 most common fractures as increasingly more patients access the Internet for medical information [31]. Specifically, when patients turn to the Internet, 92% (207.0/225.0) of them research specific medical conditions as opposed to searching for general information on healthy lifestyles. Furthermore, although there is existing literature investigating other orthopedic conditions such as femoroacetabular impingement and rotator cuff tears, there is no comprehensive study on fracture care information. As there is variability in quality within Web-based orthopedic literature and throughout nonorthopedic topics, there is a need for studies investigating specific medical conditions [32-34]. Moreover, many physicians are unaware whether or not to encourage patient use of the Internet for medical information because they do not want patients to be misinformed [35]. With a better understanding of the literature that exists for patients on fracture care, surgeons can give better “internet prescriptions,” or recommendations for improved Internet use [35]. The overall goal is for the patient to be best informed on the topic of fractures as this may lead to better self-care and improved health decisions [36].

### Key Findings and Recommendations

In this study, we found that the quality of Web-based information on the 10 most common fractures was in general “fair.”

Furthermore, there was a significant decrease in the quality of websites as the search engine user progressed to each subsequent website result on the search results page. Therefore, physicians should instruct their patients to begin their research by using the first website on the search results page and progress downward if needed.

Furthermore, the presence of a HONcode certification had a significant positive correlation with the quality of websites. As a result, physicians may inform their patients that they are more likely to find higher quality information on websites displaying a HONcode certification seal. In addition, creators of health information websites should apply for HONcode certification because following HONcode principles will likely improve the quality of their websites.

On average, the readability of all websites fell within the recommended range for the general public using the FRES formula. The FKGL was above the recommended range for the general public, however, it still fell below the cutoff for the FKGL of the average US adult, which suggests that most patients still easily understand the material.

**Figure 5:**
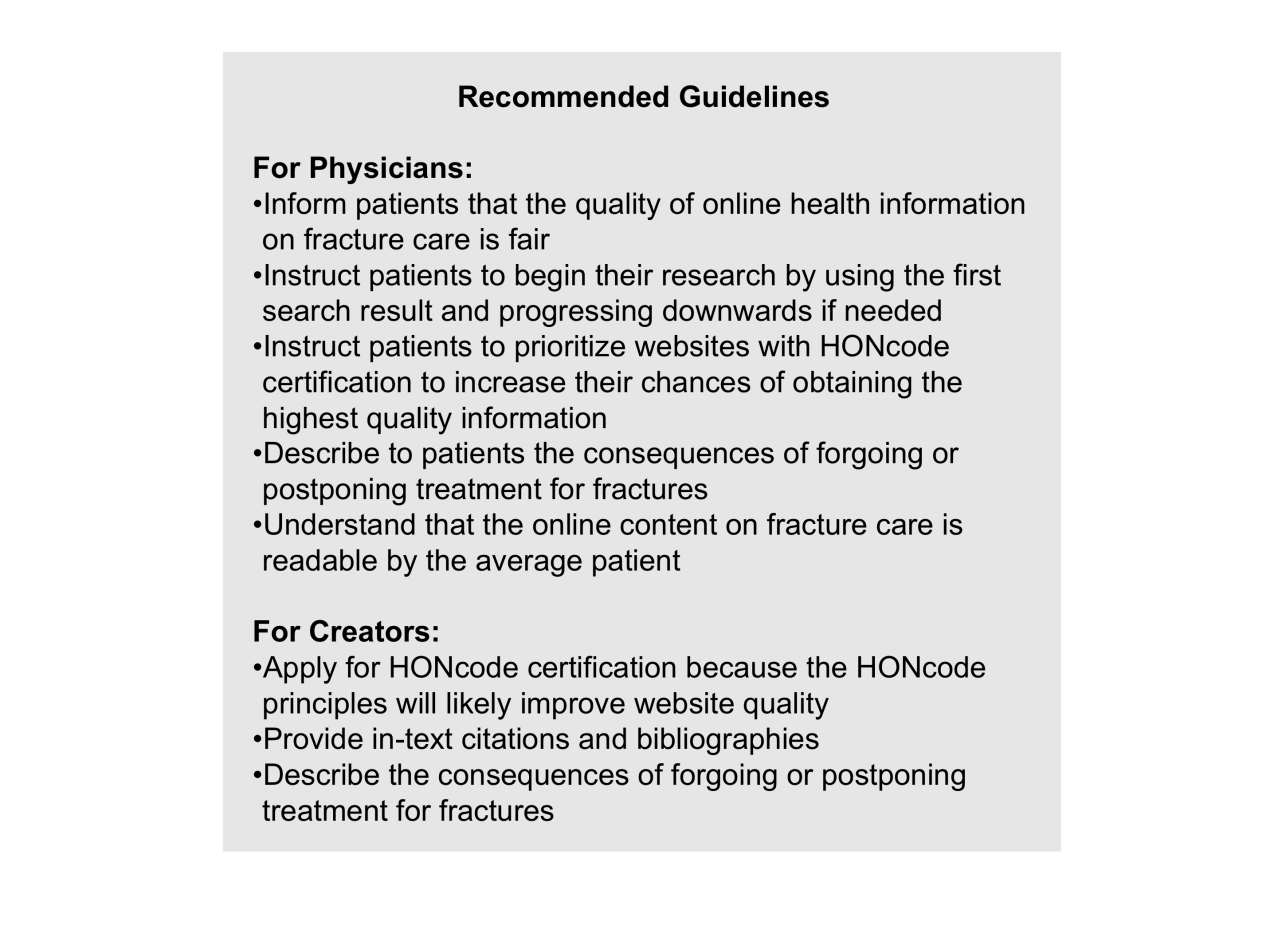
Recommended guidelines for physicians and creators of Web-based health information websites.

One question on the DISCERN instrument that was consistently answered poorly was question 4 (Is it clear what sources of information were used to compile the publication [other than the author or producer]?). As a result, health information website creators should increase the presence of in-text citations and bibliographies. Another question that was consistently answered poorly was question 12 (Does it describe what would happen if no treatment is used?). Therefore, during medical encounters, physicians should describe to their patients the consequences of forgoing or postponing treatment. Health information website creators should also provide this information on the Web. These recommendations are summarized in Figure 5, which presents a practical guideline based solely on the results of this study, aimed to assist physicians and creators of Web-based health information.

### Strengths and Limitations

This was the first study investigating the quality of Web-based health information on fracture care. Furthermore, it simulated real-world search engine usage by using the results on the first page. Many other studies have used the first 3 pages of results, which may not be representative of the search strategies used by the average search engine user and may also lower the mean quality of the results if the websites on the second and third page are of lower quality. Another reason this study is applicable is that it used the Google search engine rather than incorporating other search engines less commonly used by patients. Google is overwhelmingly the most popular search engine among patients and including other search engines in the study may have produced results that are not generalizable to a patient population [10].

There are some limitations inherent in this study. In an effort to increase the external validity of the findings by limiting the search results to the first page, one limitation was that the sample size was reduced. Second, the results were gathered at one time point and at one geographical location. In reality, search results vary over time and also vary with geographical location. Third, website names, URLs, and designs may have biased the quality assessment. A fourth limitation was that websites were excluded if they were non-English, and therefore, the results may not be applicable to a non–English-speaking population. Finally, websites that are primarily nonreadable formats (eg, video) were excluded. This may have decreased the generalizability of our results as patients may use video-sharing websites given that video-sharing websites such as YouTube are among the most visited websites worldwide.

### Implications for Future Research

It has been suggested that patients may limit themselves to using lay search terms because they are unfamiliar with orthopedic terminology [13]. However, data from the iProspect Search Engine User Behavior Study show that search engine use is dynamic and that 41% (971.0/2369) of users change their search terms if they do not find what they are looking for on their first search [37]. Therefore, patients may modify their search strategy by replacing lay search terms with newfound medical terminology. For example, a search using the term, “broken wrist” may lead them to a search using the term, “distal radius fracture.” Therefore, it would be appropriate to investigate how the quality of Web-based health information on fracture care changes with search term usage.

Furthermore, as more Web-based evidence-based materials become available for patients through UpToDate, BMJ Best Practice, Mercks Manuals, and so forth, physicians are urged to direct their patients to these resources. However, the quality and readability of these materials has not been evaluated for fracture care and reviewing these materials will help physicians make better recommendations for patients who wish to obtain information via the Internet.

### Conclusion

The quality of Web-based health information on fracture care is fair. The readability of this information is appropriate for the general public. We recommend that physicians inform their patients of the quality of Web-based health information. Furthermore, physicians should instruct their patients to select websites that have a HONcode certification seal to increase their chances of obtaining the highest quality information. Finally, physicians should instruct their patients to select websites that are positioned higher on the Google search results page because the Google ranking algorithms order the search results in a hierarchy by popularity and consequently appear to rank the websites by quality.

## Appendix

Multimedia Appendix 1 DISCERN instrument with mean score per question.

## Abbreviations

HONcode: Health on the Net code
FRES: Flesch Reading Ease Score
FKGL: Flesch-Kincaid Grade Level

## References

[1] Baker JF, Green J, Synnott KA, Mulhall K (2013). Internet use in an orthopaedic outpatient population. Curr Orthop Pract.

[2] Wetzler M (2013). “I found it on the internet:” how reliable and readable is patient information?. Arthroscopy.

[3] Fox S, Rainie L (2014). Pew Research Center.

[4] Choudhury MD, Morris MR, White RW (2014). Seeking and Sharing Health Information Online: Comparing Search Engines and Social Media. Proceedings of the SIGCHI Conference on Human Factors in Computing Systems.

[5] Aitken M (2014). Engaging patients through social media.

[6] Purcell K, Brenner J, Rainie L (2012). Search Engine Use 2012.

[7] Ahmad F, Hudak PL, Bercovitz K, Hollenberg E, Levinson W (2006). Are physicians ready for patients with Internet-based health information?. J Med Internet Res.

[8] Eysenbach G, Powell J, Kuss O, Sa E (2002). Empirical studies assessing the quality of health information for consumers on the world wide web: a systematic review. JAMA.

[9] Garcia RM, Messerschmitt PJ, Ahn NU (2009). An evaluation of information on the Internet of a new device: the lumbar artificial disc replacement. J Spinal Disord Tech.

[10] Dalton DM, Kelly EG, Molony DC (2015). Availability of accessible and high-quality information on the Internet for patients regarding the diagnosis and management of rotator cuff tears. J Shoulder Elbow Surg.

[11] Lee S, Shin JJ, Haro MS, Song SH, Nho SJ (2014). Evaluating the quality of Internet information for femoroacetabular impingement. Arthroscopy.

[12] Court-Brown CM, Caesar B (2006). Epidemiology of adult fractures: A review. Injury.

[13] McCormack D, Evoy D, Mulcahy D, Walsh M (1997). An evaluation of patients comprehension of orthopaedic terminology: implications for informed consent. J R Coll Surg Edinb.

[14] (2013). Chitika.

[15] Charnock D, Shepperd S, Needham G, Gann R (1999). DISCERN: an instrument for judging the quality of written consumer health information on treatment choices. J Epidemiol Community Health.

[16] Kincaid JP, Fishburne RP, Rogers RL, Chissom BS (1975). DTIC.

[17] Doak CC, Doak LG, Root JH (1996). Teaching patients with low literacy skills.

[18] Badarudeen S, Sabharwal S (2008). Readability of patient education materials from the American Academy of Orthopaedic Surgeons and Pediatric Orthopaedic Society of North America web sites. J Bone Joint Surg Am.

[19] (2016). AAOS.

[20] (2016). Mayo Clinic.

[21] (2016). Emedicinehealth.

[22] (2016). WebMD.

[23] (2016). WebMD Boots.

[24] (2016). Drugs.

[25] (2016). Healthline.

[26] (2016). Wikipedia.

[27] (2016). Cleveland Clinic.

[28] (2016). Physioworks.

[29] (2016). Foothealthfacts.

[30] (2016). NHS.

[31] Diaz JA, Griffith RA, Ng JJ, Reinert SE, Friedmann PD, Moulton AW (2002). Patients’ use of the internet for medical information. J Gen Intern Med.

[32] Feghhi DP, Komlos D, Agarwal N, Sabharwal S (2014). Quality of online pediatric orthopaedic education materials. J Bone Joint Surg Am.

[33] Morr S, Shanti N, Carrer A, Kubeck J, Gerling MC (2010). Quality of information concerning cervical disc herniation on the Internet. Spine J.

[34] Mathur S, Shanti N, Brkaric M, Sood V, Kubeck J, Paulino C, Merola AA (2005). Surfing for scoliosis: the quality of information available on the Internet. Spine (Phila Pa 1976).

[35] Gerber BS, Eiser AR (2001). The patient physician relationship in the Internet age: future prospects and the research agenda. J Med Internet Res.

[36] Wanless D (2002). Derechosciudadania.

[37] (2006). iProspect.

