# Supplementary figures and images for: Quality of Web-based Information for the 10 Most Common Fractures
Source: Interact J Med Res. 2016 Jun 17;5(2):e19. doi: 10.2196/ijmr.5767 (PMC4930531; doi:10.2196/ijmr.5767)

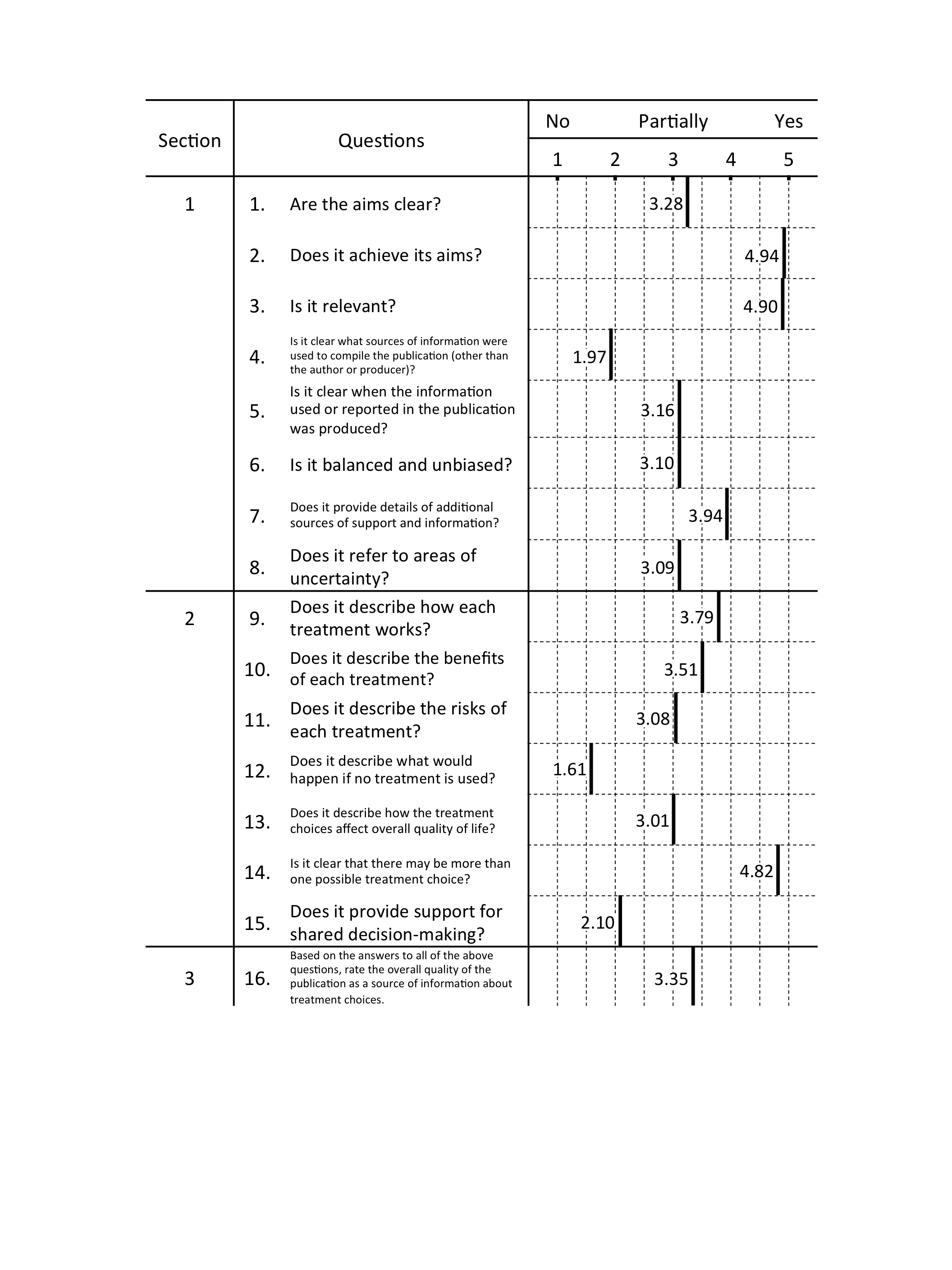

Supplement: Multimedia Appendix 1 [file ijmr_v5i2e19_app1.png]
